# Preloaded D-methionine protects from steady state and impulse noise-induced hearing loss and induces long-term cochlear and endogenous antioxidant effects

**DOI:** 10.1371/journal.pone.0261049

**Published:** 2021-12-08

**Authors:** Kathleen Campbell, Nicole Cosenza, Robert Meech, Michael Buhnerkempe, Jun Qin, Leonard Rybak, Daniel Fox

**Affiliations:** 1 Department of Medical Microbiology, Immunology, and Cell Biology, Southern Illinois University School of Medicine, Springfield, Illinois, United States of America; 2 Department of Internal Medicine, Statistics, Southern Illinois University School of Medicine, Springfield, Illinois, United States of America; 3 Department of Engineering, Southern Illinois University, Carbondale, Illinois, United States of America; 4 Department of Otolaryngology Head and Neck Surgery, Southern Illinois University School of Medicine, Springfield, Illinois, United States of America; 5 Department of Clinical Research, Springfield Clinic, Springfield, Illinois, United States of America; University of Auckland, NEW ZEALAND

## Abstract

**Objective:**

Determine effective preloading timepoints for D-methionine (D-met) otoprotection from steady state or impulse noise and impact on cochlear and serum antioxidant measures.

**Design:**

D-met started 2.0-, 2.5-, 3.0-, or 3.5- days before steady-state or impulse noise exposure with saline controls. Auditory brainstem response (ABRs) measured from 2 to 20 kHz at baseline and 21 days post-noise. Samples were then collected for serum (SOD, CAT, GR, GPx) and cochlear (GSH, GSSG) antioxidant levels.

**Study sample:**

Ten *Chinchillas* per group.

**Results:**

Preloading D-met significantly reduced ABR threshold shifts for both impulse and steady state noise exposures but with different optimal starting time points and with differences in antioxidant measures.

For impulse noise exposure, the 2.0, 2.5, and 3.0 day preloading start provide significant threshold shift protection at all frequencies. Compared to the saline controls, serum GR for the 3.0 and 3.5 day preloading groups was significantly increased at 21 days with no significant increase in SOD, CAT or GPx for any impulse preloading time point. Cochlear GSH, GSSG, and GSH/GSSG ratio were not significantly different from saline controls at 21 days post noise exposure.

For steady state noise exposure, significant threshold shift protection occurred at all frequencies for the 3.5, 3.0 and 2.5 day preloading start times but protection only occurred at 3 of the 6 test frequencies for the 2.0 day preloading start point. Compared to the saline controls, preloaded D-met steady-state noise groups demonstrated significantly higher serum SOD for the 2.5–3.5 day starting time points and GPx for the 2.5 day starting time but no significant increase in GR or CAT for any preloading time point. Compared to saline controls, D-met significantly increased cochlear GSH concentrations in the 2 and 2.5 day steady-state noise exposed groups but no significant differences in GSSG or the GSH/GSSG ratio were noted for any steady state noise-exposed group.

**Conclusions:**

The optimal D-met preloading starting time window is earlier for steady state (3.5–2.5 days) than impulse noise (3.0–2.0). At 21 days post impulse noise, D-met increased serum GR for 2 preloading time points but not SOD, CAT, or GpX and not cochlear GSH, GSSG or the GSH/GSSG ratio. At 21 days post steady state noise D-met increased serum SOD and GPx at select preloading time points but not CAT or GR. However D-met did increase the cochlear GSH at select preloading time points but not GSSG or the GSH/GSSG ratio.

## Introduction

Although physical hearing protectors have improved and many occupational noise exposures reduced, permanent noise- induced hearing loss (NIHL) still affects at least 10 million Americans [1,2]. Further, harmful levels of occupational noise exposure may affect close to 30 million Americans [3]. Approximately 37.5 million adults 18 and over report some trouble hearing [4,5], consequently NIHL would account for a large percent of the overall incidence of hearing loss in this country. Internationally, excessive noise exposure is the major avoidable cause of permanent hearing loss worldwide [6,7]. Additionally, recreational activity with firearms, amplified music, motorcycles, and power tools can expose millions of people to sound capable of producing permanent hearing loss [3,8,9]. Even young children can suffer NIHL after exposure to sudden noise emitted by toy pistols and firecrackers [10,11].

In U.S veterans, auditory disorder is the second most common service-connected injury, second only to musculoskeletal disability, and the number of new compensation recipients has grown from 250,435 in 2015 to 278, 501 in 2019 [12].

NIHL generally first affects the high frequency hearing range with a characteristic “notch” at approximately 4 kHz. As the loss progresses, the patient may have difficulty in all listening environments affecting both their social and work lives, and sometimes impacting employability. The social and psychological impact on the US population can be difficult to quantify. But the financial impact to the U.S. government is undeniable. The U.S. Veteran’s Administration alone paid approximately $24 billion dollars in hearing loss compensation from 1970–1990 [13] and $1.2 billion in 2012 [14] although the total costs to the US military are far more extensive including costs for hearing aids, retraining, noise mitigation, medical care, transportation, hearing protection devices and work time loss [15]. Noise-induced tinnitus is less well studied but is a frequently reported consequence of noise exposures [16]. The worldwide social and financial impact of military, industrial, and recreational NIHL is enormous. The World Health Organization estimates the annual global cost of unaddressed hearing loss exceeds over 750 billion dollars reported in US dollars [7].

In multiple studies, D-methionine (D-met) administered before and/or after noise exposure provides excellent noise-induced hearing loss (NIHL) protection from steady-state and impulse noise exposures [17–23]. We first reported that D-met preloading, administering D-met solely prior to noise exposure, could significantly reduce NIHL in 2013 [23]. Preloading, an innovative approach to prophylactic dosing, would be particularly useful for special operations troops that are typically deployed for 72 hours but have severe pack weight and possibly fluid restrictions during deployment. Preloading could also be useful for anticipated noise exposures such as weapons training for military and police personnel, farmers, musicians or concert attendees, or for recreational hunters and other shooters.

However, optimal preloading D-met otoprotection is time-dependent and more information is needed to optimize future clinical use. Claussen et al 2013 [23] was a small-scale study utilizing only steady state noise but many anticipated noise exposures such as weapons training are impact noise. Further we need to understand more about the mechanisms of preloading protection and whether or not long-term protective changes occur in the serum and/or cochlea.

Previous mechanistic studies cite possible correlations between D-met otoprotection and potential antioxidant enzyme biomarkers [20,24–26]. D-met is an established direct antioxidant free radical scavenger [18,20,22,26,27] and has influenced superoxide dismutase (SOD) and catalase (CAT) antioxidant biomarker levels in previous noise and drug-induced otoprotective studies [20,26]. SOD and CAT are demonstrated biomarkers for schizophrenia [28] and 3-methylglutaconic aciduria [29]. Thus, endogenous SOD and CAT enzyme assays may provide feasible and accessible biomarker opportunities to elucidate D-met’s optimal protective prophylactic dose and understand how D-met treatment influences the overall antioxidant environment.

As a sulfhydryl reversible antioxidant [30], methionine may impact the glutathione pathway ^24^, particularly in the mitochondria [31]. Although it is possible for D-met to act as a cysteine sink to fuel the glutathione pathway, methionine in its D-isomer form is excreted without conversion to the L-isomer 60–70% of the time in humans [32,33], making D-met’s direct antioxidant mechanisms the likely otoprotective pathway in humans. D-met has influenced glutathione pathway markers in previous otoprotective animal studies [25]. Further, glutathione reductase (GR) and glutathione peroxidase (GPx) are greatly influenced by NIHL [34] and are demonstrated biomarkers for GR blood deficiencies [35]. Thus, reduced oxidized glutathione ratios, particularly in cochlear supernatant, and endogenous glutathione enzymes are justified potentials as optimal D-met otoprotection biomarkers.

Combined, cochlear GSH/GSSG ratio and endogenous antioxidant assays may serve as biomarkers for optimal D-met otoprotection. They may also correlate endogenous antioxidant levels with cochlear oxidative environments and identify opportunities to develop realistically-scalable and personalized otoprotective biomarker diagnostics.

The current study identified the earliest preloaded (earliest possible time to initiate effective therapeutic prophylaxis) time possible to successfully demonstrate otoprotection from steady state or impulse noise exposures and the optimal time period for administration. The study then investigates whether or not D-met’s optimal and sub-optimal protections were also reflected in cochlear GSH/GSSG ratios and serum endogenous antioxidant enzyme levels 21 days after noise cessation.

The overall aims of the of the present study were twofold. The first aim was to determine the optimal time windows for D-met preloading administration to reduce noise-induced hearing threshold shift secondary both steady-state and impulse noise exposures. The second aim was to expand our understanding of preloaded D-met’s impact on oxidative state measured in both serum and cochleae at 21 days. These aims were achieved.

## Materials and methods

### Subjects

Study subjects comprised one hundred and ten (110) male *Chinchillas lanigera* (3–5 year of age; Ryerson, USA). Animals were housed in the SIU School of Medicine animal care facility and maintained a normal diet prior to and throughout the study’s duration. Male chinchillas are singly housed to avoid fighting. Temperature was maintained at 68 degrees Fahrenheit. Lighting was on a 12 hour on and 12 hour off cycle controlled by a timer. Animals had access to food and water ad libitum.

### Ethics statement

This study was carried out in strict accordance in the Guide for the Care and Use of Laboratory Animals of the National Institutes of Health. The protocol was approved by the Laboratory Animal Care and Use Committee (LACUC) of Southern Illinois University School of Medicine LACUC Protocol # 93-14-025.

All animal protocols and procedures were reviewed and approved by SIU School of Medicine’s Laboratory Animal Care and Use Committee prior to study performance.

### Experimental design

Subject groups (10 animals/group; 11 groups) were assigned to one of the following five (5) steady state or impulse noise exposure preloaded treatment (treatment beginning prior to noise exposure) cohorts: 2.0-day preloaded control, 3.5-day preloaded D-met, 3.0-day preloaded D-met, 2.5-day preloaded D-met, 2.0-day preloaded D-met. All groups received a total of 5 intraperitoneal (ip) injections every 12 hours for 48 hours with each dose comprising either one injection of saline (control) or one injection of 200 mg/kg D-met with start times of the first dose as indicated above. In addition, one no noise control group was sacrificed for serum enzyme and cochlear glutathione levels only to serve as a normal comparison for those measures. Auditory brainstem responses (ABRs) were measured prior to preloading treatment and noise exposure to serve as a comparative baseline and establish normal hearing thresholds, based on the lowest intensity capable of eliciting a replicable, visually detectable response in both intensity series. ABRs were measured again 21 days post-noise exposure, and threshold shift was calculated as the difference between the 21-day and baseline hearing thresholds. Animals were humanely sacrificed following 21-day ABR testing by decapitation while still under anesthesia and serum and cochlear tissues were collected for correlative enzymatic and antioxidant concentration analyses.

### D-methionine

In a sterile hood environment, 99+% pharmaceutical-grade D-methionine (Acros Organics) was dissolved into 0.9% sterile saline at 30 mg/ml. The solution was then stored in the refrigerator until use.

### Noise exposure

Steady-state and impulse noise exposures were generated by custom-developed digital noise exposure systems. Steady-state noise exposure comprised 105 dB SPL octave band noise centered at 4 kHz for a 6-hour exposure. Impulse noise exposure comprised 155 dB peak SPL impulse noise repeating 150 times over 75 seconds (simulating M-16 repeating rifle fire). Each noise exposure system comprises a data acquisition device (NI DAQUSB-6251), an audio power amplifier (Yamaha P2500S), an acoustic compression driver (JBL 2446J), a shock tube extension (3’ length and 2” diameter), an exponential horn (JBL 2380), a sound measurement and calibration system, and a computer. LabVIEW software was used to generate digital noise, control analog signal input and output, calibrate the system, and monitor sound levels during exposure experiments. The system has been described by Qin et al 2014 [36].

### Anesthesia

The injectable anesthesia solution contained 50 mg/kg ketamine and 5 mg/kg xylazine. Subjects were anesthetized via intramuscular injections prior to and during (as needed in half-original volume doses) ABR testing and prior to humane sacrifice which comprised decapitation while still under anesthesia. Throughout ABR testing, anesthetized animals were placed on a temperature-controlled thermostatic fluid pad and body temperature was monitored via rectal temperature measurements. Food, but not water, was removed from the animal cage at least 2 hours prior to anesthesia.

### Electrophysiology

ABR measurements were collected to assess bilateral auditory thresholds. Following assessment for middle ear disease, subcutaneous needle electrodes were placed into anesthetized subjects at the vertex (active electrode), nose (reference channel 1), mastoid (reference channel 2), and opposite hind leg (ground electrode). ABR thresholds were measured in response to tone bursts with 1 ms rise/fall and 0 ms plateau, gated by a Blackman envelope, and centered at 2, 4, 6, 8, 14, and 20 kHz frequencies presented at 30/s. Two intensity series ranging from 100 to 0 dB peak sound pressure level (SPL) at 512 sweeps per average were obtained for each animal in 10 dB decrements. The recording epoch was 15 ms following stimulus onset and responses were analog-filtered with a 30–3000 Hz bandpass.

### Tissue collection, preservation, and preparation

Blood samples were collected from heavily-anesthetized subjects via cardiac puncture following 21-day ABR assessment, stored in cryo vials containing 0.1mM EDTA, and immediately snap-frozen in liquid nitrogen. After blood collection, subjects were sacrificed by decapitation, right and left cochleae were collected, and samples were immediately snap-frozen in liquid nitrogen.

Prior to enzyme analysis: 1) To chelate EDTA and clot blood, 140 μL of 1 M CaCl_2_ in sterile H_2_O (Catalog no: C4901, Sigma, MO) were added to 500 μL of whole blood and the tubes were incubated on ice for 30 minutes without disturbance. After blood samples were coagulated using calcium chloride, they were then separated via centrifugation to collect serum samples; 2) The cochleae were decapsulated without the vestibulum. Cochlear tissue from the right cochlea of each animal was homogenized with a battery powered Bio-Vortexer Homogenizer rotating plastic pestles in 1.5 ml microtubes, with one cochlea per microtube in 0.1M Phosphate Buffered Saline, pH 7.4 with 0.1 mM EDTA, spun down via centrifugation. Then the supernatant was collected. for the assay.

### Enzyme assays

Serum enzyme assays were measured using an EPOCH spectrophotometer. Assays were performed in triplicate and results were normalized to total protein values via a Coomassie Plus Assay Kit (Fisher Scientific 23236). Endogenous glutathione peroxidase (GPx), glutathione reductase (GR), and superoxide dismutase (SOD) serum concentrations were quantified using manufactured kits (GPx, Cayman Chemical 703102; GR, Cayman Chemical 703202; SOD, Fisher Scientific EIASODC) per manufacturer’s instructions. Serum catalase activity was measured by previously-established H_2_O_2_ degradation protocols (Campbell et al. 2003). Briefly, 100% ethanol was added to samples at a 1:10 ratio and placed in an ice bath for 30 minutes. Following the ice bath, Triton X-100 was added to the sample at a 1:10 ratio and the resulting extract was diluted 1:25 in 0.05 M sodium phosphate buffer. Equal amounts of diluent and 0.066 M H_2_O_2_.(Sigma Catalog number 323381) were then added to initiate the quantifiable reaction. The sample was measured at 240 nm 5 times per minute for one minute and a molar extinction coefficient of 43.6 M^-1^ cm^-1^ was used to quantify catalase activity (1 unit = 1 mM H_2_O_2_ degraded/min/mg protein). A Gen5 Take3 low-volume accessory (BioTek Instruments, Inc plate was used for catalase measures only).

Cochlear supernatant samples were tested for glutathione: glutathione disulfide (GSH/GSSG) ratios using a Promega GLOMAX Multi + Detection System luminometer and a GSH/GSSG-GLO assay kit (Promega V6612). Assays were performed in duplicate and according to manufacturer’s instructions.

### Statistical analysis

Statistical analyses were performed separately for all outcomes–ABR shift, blood serum enzymes (CAT, SOD, GR, and GPx), and cochlear oxidative state (GSH, GSSG, GSH:GSSG). For the ABR shift analyses, differences in ABR threshold shift in the left and right ears were compared using paired t-tests at each frequency. No significant differences were found, and threshold shifts for the left and right ear were averaged to yield a single ABR shift for each animal. ABR threshold shift results are presented in the main text for simplicity. However, full results on baseline and 21-day post-exposure ABR shifts can be found in the supplement (S1–S4 Figs). ABR threshold shifts were analyzed using a three-way repeated measures ANOVA. Frequency (2, 4, 6, 8, 14, and 20 kHz) was included as a repeated within-subjects factor. Treatment group (saline and D-met preloading at 2, 2.5, 3, and 3.5 days) and noise exposure (impulse and steady-state) were included as between-subject factors. Post-hoc contrasts explored all pairwise differences between treatment groups at each level of frequency and noise exposure with p-values adjusted for multiple comparisons using Tukey’s method.

Each blood serum enzyme measure and cochlear oxidative state measure was analyzed using a two-way ANOVA. Treatment group (saline and D-met preloading at 2, 2.5, 3, and 3.5 days) and noise exposure (impulse and steady-state) were included as between-subject factors. Post-hoc contrasts explored all pairwise differences between all treatment groups and saline controls at each level of noise exposure with p-values adjusted for multiple comparisons using Tukey’s method. Additional one-way ANOVAs were used to explore differences between the no noise control group and treatment groups under each noise exposure. Post-hoc contrasts explored the difference between each treatment group and the no noise controls with p-values adjusted to correct for multiple comparisons using Tukey’s method.

## Results

### ABR threshold shift

ABR threshold shifts varied significantly across treatment group (p < 0.001) but not across noise exposure (p = 0.20) or frequency (p = 0.09). A significant interaction existed between treatment group and noise exposure (p < 0.001). Other two-way interactions (noise exposure by frequency–p = 0.92; treatment group by frequency–p = 0.65) and the three-way interaction (p = 0.35) were not significant.

**Fig 1:**
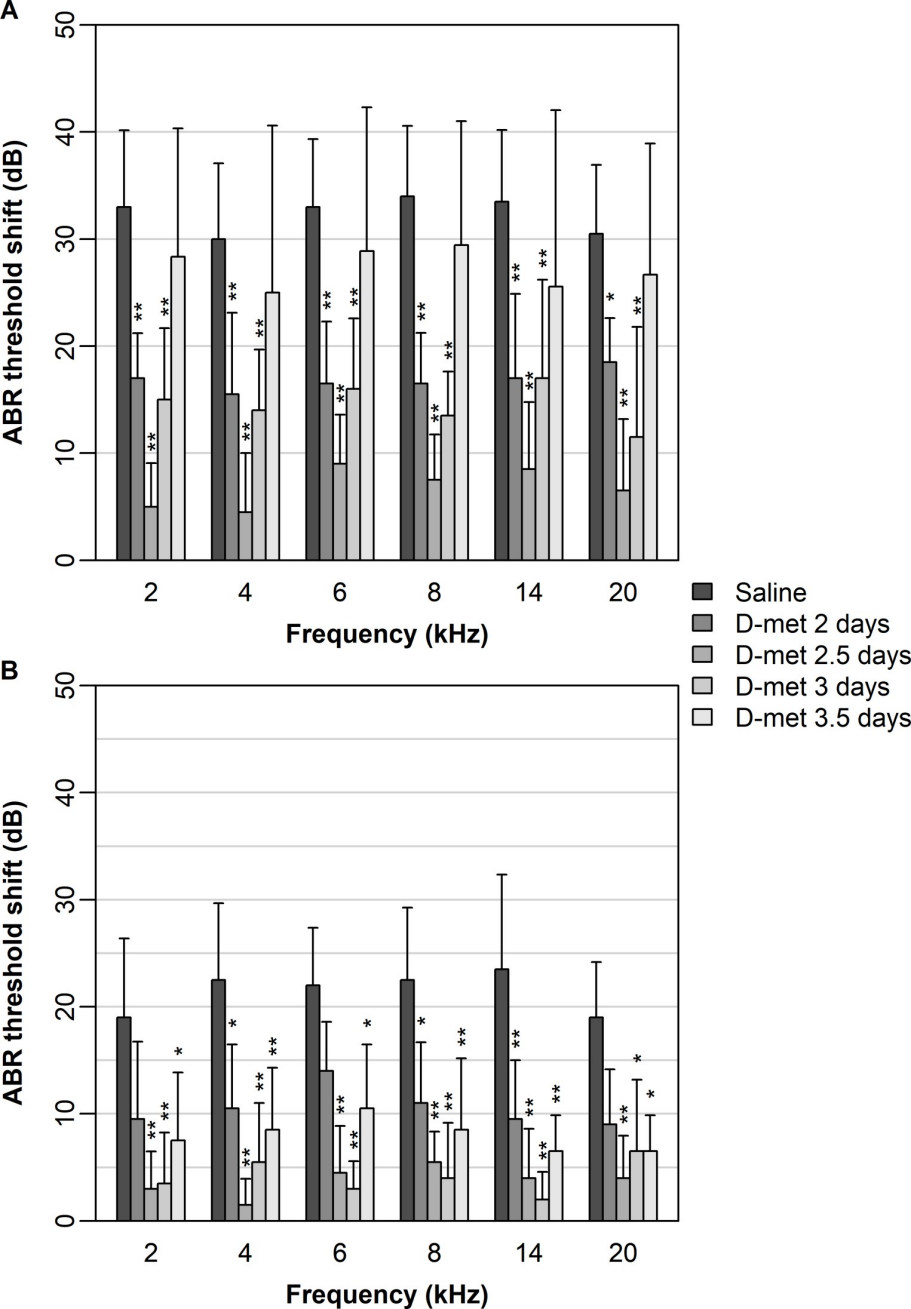
Describes the mean (± one standard deviation) ABR shift (in dB) observed at different frequencies (2, 4, 6, 8, 14, and 20 kHz) under impulse (A) and steady-state noise exposure. Results for each D-met pre-loading treatment are shown for each noise exposure–frequency combination (ranging from saline treatment [dark gray] to pre-loading at 3.5 days [light gray]). Significant differences between D-met preloading times and the saline treatment for each noise exposure–frequency combination are denoted by ‘*’ (p < 0.05) and ‘**’ (p < 0.01). Ten animals were tested in each group except for D-met preloaded at 3.5 days and exposed to impulse noise, where 9 animals were successfully tested.

For impulse noise exposure, preloading with D-met at 2, 2.5, and 3 days (but not 3.5 days) resulted in significantly lower ABR threshold shifts than saline controls across all frequencies (Fig 1A). A general trend existed across all frequencies toward the lowest ABR threshold shifts occurring when preloading with D-met at 2.5 days, although this treatment was only significantly lower than preloading at 2 and 3 days at frequencies of 2 and 4 kHz. At frequencies of 8 and 20 kHz, preloading at 2.5 days was similar to preloading at 3 days.

For steady-state noise exposure, preloading with D-met at 2.5, 3, and 3.5 days resulted in significantly lower ABR threshold shifts than the saline controls across all frequencies (Fig 1B). Preloading at 2 days resulted in significantly lower ABR threshold shifts than saline controls only at frequencies of 4, 8, and 14 kHz. Although preloading at 2.5 and 3 days generally resulted in the lowest ABR shifts, these treatments were not statistically lower than each other or preloading at 2 or 3.5 days at any frequency considered.

Thus, the effective window of preloading timepoints for D-met protection appear to extend earlier for impulse noise than for steady state noise exposures which extends later although effective time periods partially overlap.

### Serum enzymes

**Fig 2:**
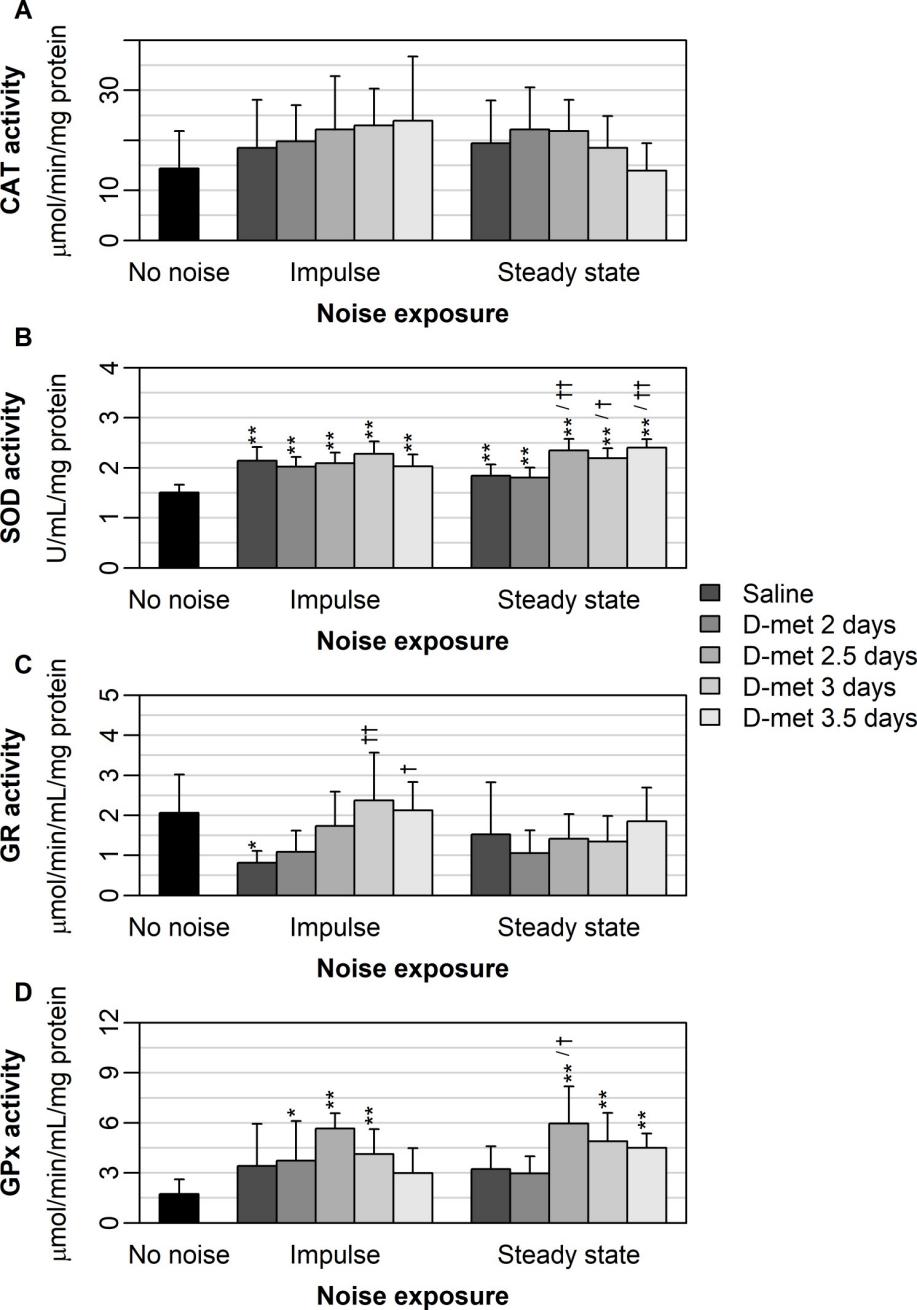
Describes the mean (± one standard deviation) serum enzyme activity under impulse and steady-state noise exposure. Serum enzymes analyzed included: CAT (A), SOD (B), GR (C), and GPx (D). Results for each D-met pre-loading treatment are shown for each noise exposure (ranging from saline treatment [dark gray] to pre-loading at 3.5 days [light gray]). No noise controls are shown in black. Significant differences between D-met pre-loading times and no noise controls for each noise exposure are denoted by ‘*’ (p < 0.05) and ‘**’ (p < 0.01). Significant differences between D-met pre-loading times and the saline treatment for each noise exposure are denoted by ‘†’ (p < 0.05) and ‘††’ (p < 0.01). Ten animals were tested in each group.

CAT activity showed no significant difference across treatment group (p = 0.75) or no noise exposure (p = 0.19), and no significant treatment by noise interaction was found. CAT activity was similar between all treatment groups, saline controls, and no noise controls under impulse and steady-state noise exposure (Fig 2A).

SOD activity varied significantly across treatment groups (p < 0.001) but not across noise exposure type (p = 0.89). A significant noise exposure by treatment group interaction (p < 0.001) existed. SOD activity was similar between treatment groups and saline controls under impulse noise exposure, and all treatment groups had higher SOD activity than the no noise controls (Fig 2B). No significant difference was found between treatment groups for impulse noise exposure, however. For steady-state noise exposure, preloading at 2.5, 3, and 3.5 days showing significantly higher activity than saline treatment (Fig 2B). All treatment groups had higher SOD activity than no noise controls (Fig 2B), and preloading at 2.5, 3, and 3.5 days showed significantly higher activity than preloading at 2 days.

GR activity varied significantly across treatment groups (p = 0.002) but not across noise exposure (p = 0.26). A significant noise exposure by treatment group interaction (p = 0.04) was found. For impulse noise exposure, preloading at 3 and 3.5 days yielded significantly higher GR activity than saline controls (Fig 2C). Saline controls had significantly lower GR activity than no noise controls, while all other treatments were similar to the no noise controls (Fig 2C). Preloading at 3 and 3.5 days was not significantly different from other preloading times. For steady-state noise exposure, GR activity was similar between saline controls, no noise controls, and all treatment groups Fig 2C).

GPx activity varied significantly across treatment groups (p < 0.001) but not across noise exposure (p = 0.35). No significant noise exposure by treatment group interaction (p = 0.28) existed. Although GPx activity was similar across saline controls and all treatment groups under impulse noise exposure, only preloading at 2, 2.5, and 3 days yielded significantly higher GPx activity than the no noise controls (Fig 2D). Under steady-state noise exposure, only preloading at 2.5 days was significantly higher than the saline controls (Fig 2D). Preloading at 2.5, 3, and 3.5 days yielded significantly higher GPx activity than the no noise controls (Fig 2D). Preloading at 2.5 days yielded significantly higher GPx than preloading at 2 days but was similar to other preloading times.

Under impulse noise exposure, only GPx activity had moderate negative correlation with observed ABR threshold shifts (2 kHz–*r* = -0.38; 4 kHz–*r* = -0.43; 6 kHz–*r* = -0.38; 8 kHz–*r* = -0.49; 14 kHz–*r* = -0.41; 20 kHz–*r* = -0.48; S1 Table). However, under steady-state noise exposure, both SOD (2 kHz–*r* = -0.29; 4 kHz–*r* = -0.38; 6 kHz–*r* = -0.48; 8 kHz–*r* = -0.38; 14 kHz–*r* = -0.47; 20 kHz–*r* = -0.37; S2 Table) and GPx were moderately, negatively correlated with ABR threshold shifts (2 kHz–*r* = -0.32; 4 kHz–*r* = -0.36; 6 kHz–*r* = -0.53; 8 kHz–*r* = -0.30; 14 kHz–*r* = -0.33; 20 kHz–*r* = -0.39; S2 Table).

### Cochlear oxidative state

**Fig 3:**
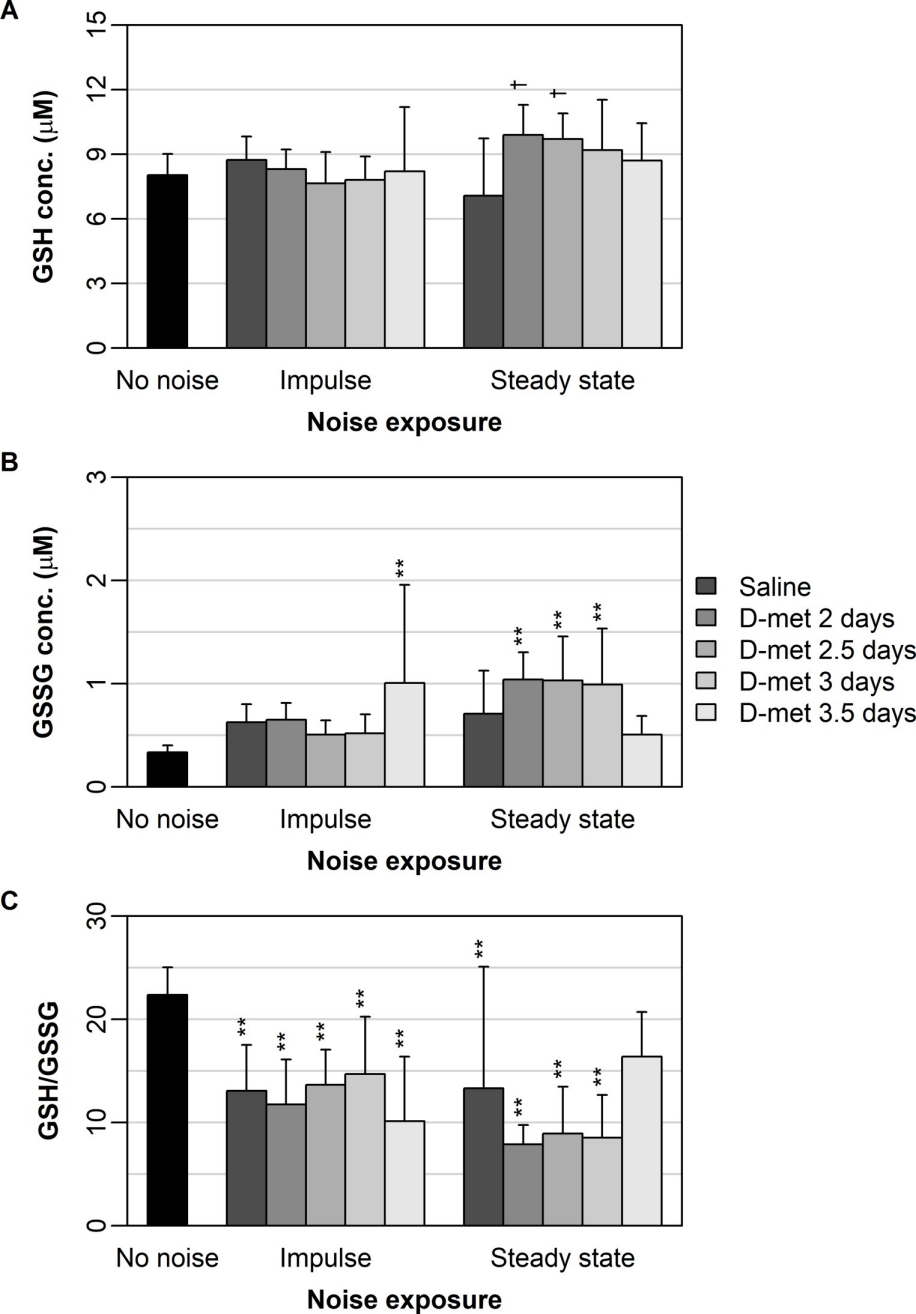
Describes the mean (± one standard deviation) of cochlear oxidative state measures under impulse and steady-state noise exposure. Cochlear oxidative state measures analyzed included: GSH (A), GSSG (B), and GSH/GSSG ratio (C). Results for each D-met pre-loading treatment are shown for each noise exposure (ranging from saline treatment [dark gray] to pre-loading at 3.5 days [light gray]). No noise controls are shown in black. Significant differences between D-met pre-loading times and no noise controls for each noise exposure are denoted by ‘*’ (p < 0.05) and ‘**’ (p < 0.01). Significant differences between D-met pre-loading times and the saline treatment for each noise exposure are denoted by ‘†’ (p < 0.05) and ‘††’ (p < 0.01).

GSH concentration varied significantly over noise exposure (p = 0.04), but not over treatment group (p = 0.34). A significant noise by treatment interaction (p = 0.01) was found. For impulse noise exposure, all treatment groups were similar and did not differ from saline and no noise controls (Fig 3A). For steady-state noise exposure, preloading at 2 and 2.5 days increased GSH above saline treatment (Fig 3A), although these two treatments produced statistically similar GSH concentrations to preloading at 3 and 3.5 days. No noise controls were similar to all treatment groups (Fig 3A).

GSSG concentration varied over noise exposure type (p = 0.02) but not over treatment group (p = 0.76). There was a significant interaction between noise and treatment (p = 0.001). For impulse noise exposure, treatment groups were similar to saline controls and each other, and only preloading at 3.5 days resulted in significantly higher GSSG concentration than no noise controls (Fig 3B). For steady-state noise exposure, all treatment groups and saline controls were similar each other, and preloading at 2, 2.5, and 3 days resulted in significantly higher GSSG concentration than no noise controls (Fig 3B).

No effect of noise exposure type (p = 0.15) or treatment group (p = 0.29) on GSH:GSSG ratio was found, but a significant interaction between noise type and treatment (p = 0.01) existed. For impulse noise exposure, no treatments were different from saline controls, but all treatments resulted in significantly lower GSH/GSSG ratios than no noise controls (Fig 3C). For steady-state noise exposure, all treatment groups were similar to saline controls, and all treatment groups, except preloading at 3.5 days, resulted in significantly lower GSH/GSSG ratios than no noise controls (Fig 3C).

The GSH:GSSG ratio was not correlated with ABR threshold shifts under impulse (S1 Table) and steady-state noise exposure (S2 Table). The GSH:GSSG ratio had a moderate inverse relationship with SOD (impulse–*r* = -0.46; steady-state–*r* = -0.43; S1 and S2 Tables) and GPx (impulse–*r* = -0.29; steady-state–*r* = -0.35; S1 and S2 Tables).

## Discussion

Prevention of NIHL could be a far more effective approach than hearing aids and disability payments in both social and economic terms [12,15]. Preloading of an otoprotective agent may afford us that opportunity, at least for anticipated noise exposures such as weapons training. However, while the military and shooters are potentially large patient populations for pre-loading, pre-loading could be useful for any anticipated high noise level exposures such as jack-hammer use and other high noise equipment, musicians, concert attendees, and firework displays. Just as people plan ahead by carrying physical hearing protectors, a pre-loading pharmacologic agent may also be helpful, particularly if one can be developed that could be delivered orally and with a suitable risk/benefit ratio.

To address the range of clinical dosing protocols that will be needed to reduce the burden of NIHL, a variety of treatment approaches will be needed. While pre-loading could be very useful for anticipated noise exposures just as are hearing protection devices, not all exposures are expected and even when they are expected not all patients may access protection in advance of the exposure. Consequently, rescue treatment ie providing the pharmacologic agent after the noise exposure and probably after a change in hearing has been noticed, is another needed approach. We have previously published 2 studies demonstrating that D-met can first be administered 1 to 7 hours post noise and significantly reduce NIHL [18,19]. Further 3 clinical trials using iv or intratympanic rescue dosing with other agents demonstrated some efficacy against NIHL following either military or firework exposure [37–39]. One challenge for rescue dosing is that the agent would need to be readily available after an unexpected exposure while preloading dosing can be planned in advance.

Pre-loading could be useful for a variety of anticipated noise exposures. However first we need to know the effective time-points of preloading and understand some of its effects on both serum and cochlear oxidative levels.

We have previously reported that in small groups of animals (n = 5 per cell) D-met preloading significantly reduced steady- state noise-induced ABR threshold shift when started 3 days prior to noise exposure [23]. However, using larger animal groups (n = 10 per cell) and more timepoints in the current study, we have found that the effective time window for D-met preloading for steady-state noise exposure actually extends from 2.5 to 3.5 days for protection across all ABR frequencies with protection at 3/6 frequencies at 2.0 day start time. Thus, the time window of effective start times for D-met preloading is wider than originally predicted. The wider range of start times could be clinically useful in allowing more flexibility for clinical use.

Because D-met preloading to reduce impulse noise-induced ABR threshold shift has not been previously investigated, we cannot compare these current to previous findings for impulse noise. The effective time window for preloading start times was earlier than for steady-state noise although overlap existed in the effective time frames. For impulse noise exposure, preloading with D-met at 2, 2.5, and 3 days (but not 3.5 days) resulted in significantly lower ABR threshold shifts than saline controls across all frequencies. Thus 2.5 and 3.0 day start times yielded optimal protection for both steady-state and impulse noise exposures but 2.0 day start time was effective at all frequencies tested for impulse noise exposures but only partially for steady-state noise exposures and the 3.5 start time was effective for steady-state noise exposures but not for impulse noise exposures.

Thus, the effective windows of preloading timepoints for D-met protection appear to extend earlier for impulse noise than for steady state noise exposures which extends later. However effective time periods partially overlap for both types of noise exposure. Further D-met’s overall mitigation of threshold shift appears to be less for impulse noise exposure than for steady state noise exposure.

Differences in damage and opportunities for protection between impulse and steady state noise exposure were expected. As reviewed by Henderson and Hamernik 1995 [40], steady state noise exposures would be expected to cause overstimulation of the outer sensory hair cells with high energy demands from mitochondrial transport chains. The more intense impulse noise exposures, would be expected to induce not only metabolic damage with outer hair cell loss but mechanical damage including tears in the basilar membrane and reticular lamina, breakage of the stereocilia tips, and even “potassium poisoning” secondary to endolymphatic infiltration into supporting cells quickly activating programmed cell death pathways [41,42]. Consequently, an antioxidant, such as D-met, may have a greater opportunity to mitigate the metabolic damage secondary to steady state noise, which could deplete endogenous antioxidants but without direct mechanical damage, as opposed to the intense damage of impulse noise which could induce not only metabolic damage but mechanical damage with higher variability, and abrupt programmed cell death activation. Thus, steady state vs. impulse noise exposures may have different antioxidant profile needs, opportunities for protection, and different outcomes.

Further D-met preloading resulted in increased serum and cochlear antioxidant levels even 21 days after noise cessation and even longer after cessation of the D-met administration which occurred prior to noise onset. D-met’s increase in GR and GPX activity is consistent with our previous study results demonstrating that D-met can protect antioxidant enzymes [25]. The increase in GPX activity observed here was associated with lower ABR threshold shifts (S1 and S2 Tables). Methionine can enhance glutathione synthesis and concentrations, particularly in the mitochondria [22,43,44], and D-met’s long-term influence on glutathione concentrations at 21 days post noise in this study provides another timepoint of information suggesting long term protection consistent with previous studies citing significant glutathione increases 2–4 months after glutathione supplementation [45].

D-met increased endogenous serum antioxidant levels just as it has in previous rescue studies [20,25,26]. This study, however, comprised a preloaded experimental design and therefore further elucidates D-met’s long-term protective characteristics. Furthermore, the increases in serum antioxidant levels, specifically GPX under impulse noise and SOD and GPX under steady-state noise, were associated with lower ABR threshold shifts in this study.

D-met also increased cochlear GSH concentrations in this preloaded study design just as it has in previous rescue studies [18,25], which may justify noted apoptosis, 4-HNE, connexin 26, and connexin 30 inhibitions when it is administered before or after noise exposure [46]. We did not observe any association between GSH:GSSG ratios and ABR threshold shifts, but we did find a negative association with serum enzyme activity, specifically SOD and GPX (S1 and S2 Tables). More research is needed to elucidate the exact relationship between functional hearing thresholds and serum enzyme and cochlear oxidative state measures. It is possible that earlier measures in antioxidant status following D-met preloading would provide even a clearer correlation between protection from noise-induced threshold shift and D-met’s antioxidant effects.

The current study did find changes in antioxidant levels 21 days post noise at the time of animal sacrifice which is encouraging for longer term protective effects. However, changes at 21 days cannot fully address the timing of optimal and suboptimal preloading timepoints for either steady state or impulse noise exposures. For example, the 3.5 day pre-loading time point, did not provide significant protection from noise-induced threshold shift for impulse noise but did for steady state noise. Further apparent preloading “sweet spots” exist in the protection for the 2.5 day start time for impulse noise and the 2.5 and 3.0 day start times for steady state noise. The lack of significant protection for impulse noise at 3.5 days when significant protection for steady state noise for the same timing may simply reflect the greater damage inflicted by impulse noise as discussed above. But the “sweet spots” of timing may be more complex. They cannot be explained simply on the basis of D-met half-life which has been reported in the rat as .9 hours [47], and 3.2 hours in humans [48] although Alagic et al 2011 [49] did demonstrate elevated cochlear D-met levels 24 hours after round window administration. If protection was solely dependent on D-met concentration at the time of noise exposure, the greatest protection would be expected to occur for the 2.0 day preloading start time that completed the 48 hours of dosing just before noise exposure, which was not the case.

Therefore the “sweet spots” are more likely secondary to D-met induced changes in antioxidant levels over time. Although the temporal antioxidant changes for the first few days following D-met administration have not been fully characterized in the literature, the information available does suggest that possibility. A single IV 50 mg/kg D-met increased cochlear reduced glutathione levels and the GSH/GSSG ratio at 4–8 hours and 4 hours respectively [50]. Samson et al 2008 [20], administered one dose of D-met prior to noise exposure and two after noise and reported the D-met to significantly ameliorate the noise induced changes in SOD levels on post-noise day 7 and CAT levels post noise days 3 and 7. Similarly, administering one pre- and two post-noise exposure D-met doses elevated CAT and SOD levels from 7–14 days peaking 7 days post-noise exposure [51]. Further studies are needed to elucidate the exact time course of preloading D-met induced changes in antioxidant status.

## Conclusions

Preloaded D-met between is optimal when started 2.0, 2.5 or 3 days prior to impulse noise exposure and 2.5, 3.0 or 3.5 days for steady state noise exposure thus the most effective time window appears to be somewhat later for impulse than for steady state noise exposures. However, effective preloading timepoints for D-met protection overlap across noise exposure types and although extending earlier for impulse noise than for steady state noise exposures which extend later. D-met also elucidates long-term cochlear and endogenous antioxidant increases up to 22.5 days post-dose and 21 days post-noise cessation, which may eventually be used to clinically-tailor optimal D-met otoprotective doses at an individualized level. Results demonstrate preloaded D-met’s protective effects from impulse noise exposure, extend protection dosing windows for steady state noise exposure, and identify potential long-term cochlear and serum antioxidant biomarkers of D-met protection. Future studies may further elucidate optimal D-met protection with additional antioxidant biomarkers, particularly at earlier timepoints.

## Supporting information

S1 Fig Mean (± one standard deviation) baseline and final ABR thresholds (dB) at frequencies of 2 (A), 4 (B), and 6 (C) kHz under impulse noise exposure. Baseline (dark gray) and final (light gray) thresholds are shown for the control group (labelled ‘Sal.’) and all D-met preloading groups (2, 2.5, 3, and 3.5 days). Ten animals were tested in each group with the exception of the D-met 3.5 day preload group where 9 animals were successfully tested.
(DOCX)

S2 Fig Mean (± one standard deviation) baseline and final ABR thresholds (dB) at frequencies of 8 (A), 14 (B), and 20 (C) kHz under impulse noise exposure. Baseline (dark gray) and final (light gray) thresholds are shown for the control group (labelled ‘Sal.’) and all D-met preloading groups (2, 2.5, 3, and 3.5 days). Ten animals were tested in each group with the exception of the D-met 3.5 day preload group where 9 animals were successfully tested.
(DOCX)

S3 Fig Mean (± one standard deviation) baseline and final ABR thresholds (dB) at frequencies of 2 (A), 4 (B), and 6 (C) kHz under steady-state noise exposure. Baseline (dark gray) and final (light gray) thresholds are shown for the control group (labelled ‘Sal.’) and all D-met preloading groups (2, 2.5, 3, and 3.5 days). Ten animals were tested in each group.
(DOCX)

S4 Fig Mean (± one standard deviation) baseline and final ABR thresholds (dB) at frequencies of 8 (A), 14 (B), and 20 (C) kHz under steady-state noise exposure. Baseline (dark gray) and final (light gray) thresholds are shown for the control group (labelled ‘Sal.’) and all D-met preloading groups (2, 2.5, 3, and 3.5 days). Ten animals were tested in each group.
(DOCX)

S1 Table Pearson correlation coefficients for ABR threshold shifts, serum enzyme activity, and cochlear oxidative state for all animals exposed to impulse noise. (DOCX)

S2 Table Pearson correlation coefficients for ABR threshold shifts, serum enzyme activity, and cochlear oxidative state for all animals exposed to steady-state noise. (DOCX)
