# Supplementary material for: Preloaded D-methionine protects from steady state and impulse noise-induced hearing loss and induces long-term cochlear and endogenous antioxidant effects
Source: PLoS One. 2021 Dec 8;16(12):e0261049. doi: 10.1371/journal.pone.0261049 (PMC8654202; doi:10.1371/journal.pone.0261049)
Supplement: S1 Fig — Mean (± one standard deviation) baseline and final ABR thresholds (dB) at frequencies of 2 (A), 4 (B), and 6 (C) kHz under impulse noise exposure. Baseline (dark gray) and final (light gray) thresholds are shown for the control group (labelled ‘Sal.’) and all D-met preloading groups (2, 2.5, 3, and 3.5 days). Ten animals were tested in each group with the exception of the D-met 3.5 day preload group where 9 animals were successfully tested. (DOCX) [file pone.0261049.s001.docx]

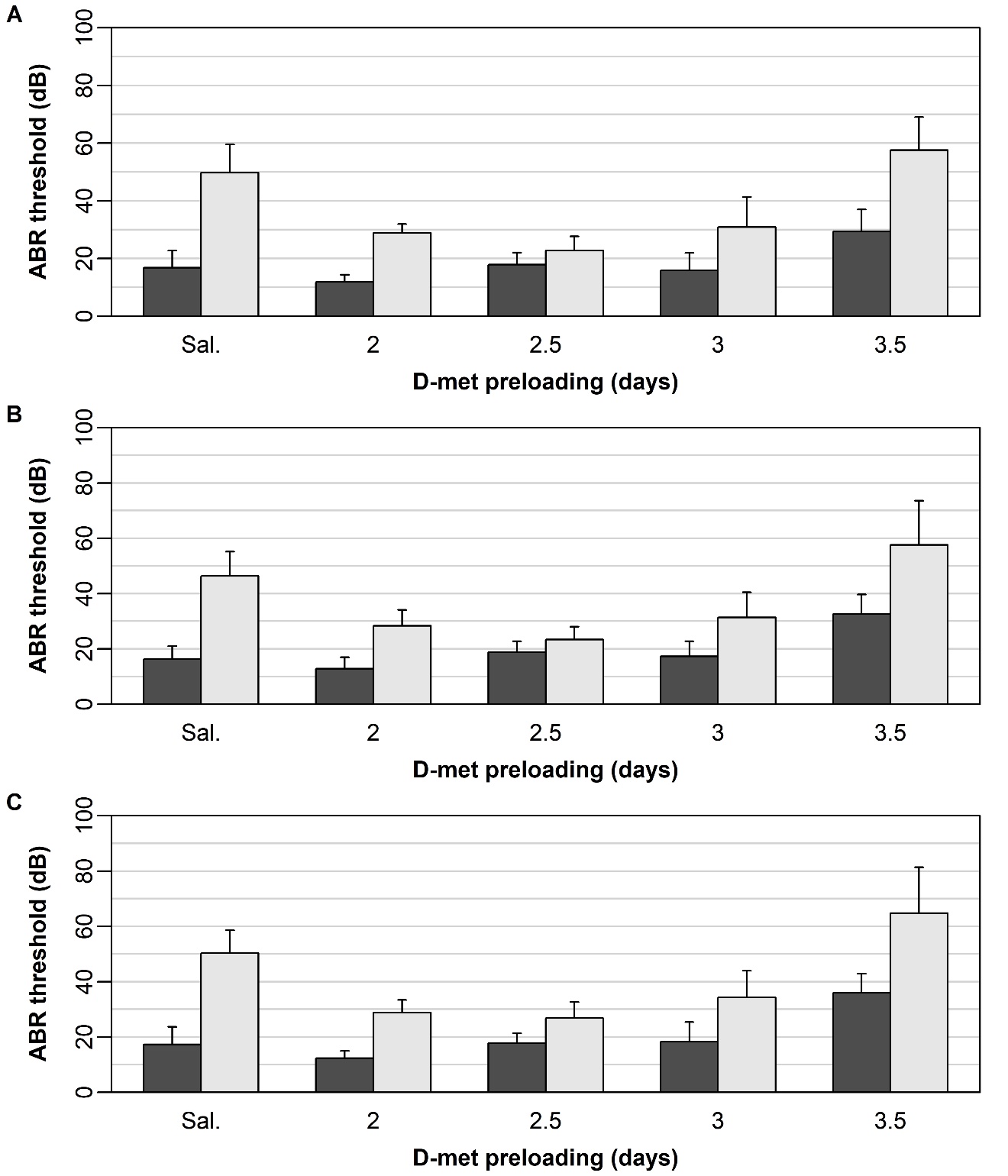


**Figure S1**: Mean (± one standard deviation) baseline and final ABR thresholds (dB) at frequencies of 2 (A), 4 (B), and 6 (C) kHz under impulse noise exposure. Baseline (dark gray) and final (light gray) thresholds are shown for the control group (labelled ‘Sal.’) and all D-met preloading groups (2, 2.5, 3, and 3.5 days). Ten animals were tested in each group with the exception of the D-met 3.5 day preload group where 9 animals were successfully tested.
