# Supplementary material for: Preloaded D-methionine protects from steady state and impulse noise-induced hearing loss and induces long-term cochlear and endogenous antioxidant effects
Source: PLoS One. 2021 Dec 8;16(12):e0261049. doi: 10.1371/journal.pone.0261049 (PMC8654202; doi:10.1371/journal.pone.0261049)
Supplement: S1 Table — (DOCX) [file pone.0261049.s005.docx]

**Table S1:** Pearson correlation coefficients for ABR threshold shifts, serum enzyme activity, and cochlear oxidative state for all animals exposed to impulse noise.

|  |  | ***ABR threshold shift*** | | | | | | ***Serum enzyme activity*** | | | | ***Cochlear oxidative state*** | | |
| --- | --- | --- | --- | --- | --- | --- | --- | --- | --- | --- | --- | --- | --- | --- |
|  |  | *2kHz* | *4kHz* | *6kHz* | *8kHz* | *14kHz* | *20kHz* | *CAT* | *SOD* | *GR* | *GPx* | *GSH* | *GSSG* | *GSH/GSSG* |
| **ABR threshold shift** | *2kHz* | 1 | 0.86 | 0.81 | 0.85 | 0.78 | 0.84 | -0.09 | -0.15 | -0.06 | -0.38 | 0.08 | 0.15 | -0.16 |
|  | *4kHz* |  | 1 | 0.80 | 0.78 | 0.82 | 0.79 | -0.15 | -0.03 | -0.10 | -0.43 | -0.05 | 0.01 | -0.12 |
|  | *6kHz* |  |  | 1 | 0.84 | 0.76 | 0.79 | -0.07 | -0.12 | -0.11 | -0.38 | -0.09 | -0.01 | -0.07 |
|  | *8kHz* |  |  |  | 1 | 0.80 | 0.81 | 0.00 | 0.00 | -0.08 | -0.49 | 0.12 | 0.16 | -0.07 |
|  | *14kHz* |  |  |  |  | 1 | 0.84 | 0.04 | -0.03 | -0.01 | -0.41 | 0.08 | 0.12 | -0.08 |
|  | *20kHz* |  |  |  |  |  | 1 | -0.10 | -0.24 | -0.10 | -0.48 | 0.14 | 0.21 | -0.19 |
| **Serum enzyme activity** | *CAT* |  |  |  |  |  |  | 1 | 0.40 | 0.13 | 0.12 | -0.14 | 0.14 | -0.22 |
|  | *SOD* |  |  |  |  |  |  |  | 1 | -0.02 | 0.31 | 0.02 | 0.22 | -0.46 |
|  | *GR* |  |  |  |  |  |  |  |  | 1 | -0.04 | 0.08 | 0.01 | 0.20 |
|  | *GPx* |  |  |  |  |  |  |  |  |  | 1 | -0.03 | 0.06 | -0.29 |
| **Cochlear oxidative state** | *GSH* |  |  |  |  |  |  |  |  |  |  | 1 | 0.62 | -0.12 |
|  | *GSSG* |  |  |  |  |  |  |  |  |  |  |  | 1 | -0.68 |
|  | *GSH/*  *GSSG* |  |  |  |  |  |  |  |  |  |  |  |  | 1 |
